# Regional [^18^F]flortaucipir PET is more closely associated with disease severity than CSF p-tau in Alzheimer’s disease

**DOI:** 10.1007/s00259-020-04758-2

**Published:** 2020-04-14

**Authors:** Emma E. Wolters, Rik Ossenkoppele, Sander C. J. Verfaillie, Emma M. Coomans, Tessa Timmers, Denise Visser, Hayel Tuncel, Sandeep S. V. Golla, Albert D. Windhorst, Ronald Boellaard, Wiesje M. van der Flier, Charlotte E. Teunissen, Philip Scheltens, Bart N. M. van Berckel

**Affiliations:** 1 Department of Radiology and Nuclear Medicine, Amsterdam Neuroscience, Vrije Universiteit Amsterdam, Amsterdam UMC, Amsterdam, The Netherlands; 2 Alzheimer Center Amsterdam, Department of Neurology, Amsterdam Neuroscience, Vrije Universiteit Amsterdam, Amsterdam UMC, Amsterdam, The Netherlands; 3 Clinical Memory Research Unit, Lund University, Lund, Sweden; 4 Department of Epidemiology and Biostatistics, Vrije Universiteit Amsterdam, Amsterdam UMC, Amsterdam, The Netherlands; 5 Neurochemistry Laboratory, Department of Clinical Chemistry, Vrije Universiteit Amsterdam, Amsterdam UMC, Amsterdam, The Netherlands

**Keywords:** [^18^F]flortaucipir, PET, CSF, Tau, Cognition, Atrophy

## Abstract

**Purpose:**

In vivo Alzheimer’s disease (AD) biomarkers for tau pathology are cerebrospinal fluid (CSF) phosphorylated tau (p-tau) and [^18^F]flortaucipir positron emission tomography (PET). Our aim was to assess associations between CSF p-tau with [^18^F]flortaucipir PET and the associations of both tau biomarkers with cognition and atrophy.

**Methods:**

We included 78 amyloid positive cognitively impaired patients (clinical diagnoses mild cognitive impairment (MCI, *n* = 8) and AD dementia (*n* = 45) and 25 cognitively normal subjects with subjective cognitive decline (SCD) (40% amyloid-positive)). Dynamic 130 min [^18^F]flortaucipir PET scans were acquired to generate binding potential (BP_ND_) images using receptor parametric mapping and standardized uptake values ratios of 80–100 min (SUVr_80-100min_) post injection. We obtained regional BP_ND_ and SUVr from entorhinal, limbic, and neocortical regions-of-interest (ROIs), closely aligning to the neuropathological tau staging schemes. Cognition was assessed using MMSE and composite scores of four cognitive domains, and atrophy was measured using gray matter volume covering the major brain lobes. First, we used linear regressions to investigate associations between CSF p-tau (independent variable) and tau PET (dependent variable). Second, we used linear regressions to investigate associations between CSF p-tau, tau PET (separate independent variables, model 1), and cognition (dependent variable). We then assessed the independent effects of CSF p-tau and tau PET on cognition by simultaneously adding the other tau biomarker as a predictor (model 2). Finally, we performed the same procedure for model 1 and 2, but replaced cognition with atrophy. Models were adjusted for age, sex, time lag between assessments, education (cognition only), and total intracranial volume (atrophy only).

**Results:**

Higher [^18^F]flortaucipir BP_ND_ was associated with higher CSF p-tau (range of standardized betas (sβ) across ROIs, 0.43–0.46; all *p* < 0.01). [^18^F]flortaucipir BP_ND_ was more strongly associated with cognition and atrophy than CSF p-tau. When [^18^F]flortaucipir BP_ND_ and CSF p-tau were entered simultaneously, [^18^F]flortaucipir BP_ND_ (range sβ = − 0.20 to – 0.57, all *p* < 0.05) was strongly associated with multiple cognitive domains and atrophy regions. SUVr showed comparable results to BP_ND_.

**Conclusion:**

Regional [^18^F]flortaucipir BP_ND_ correlated stronger with cognition and neurodegeneration than CSF p-tau, suggesting that tau PET more accurately reflects disease severity in AD.

**Electronic supplementary material:**

The online version of this article (10.1007/s00259-020-04758-2) contains supplementary material, which is available to authorized users.

## Introduction

Alzheimer’s disease (AD) is characterized neuropathologically by depositions of amyloid plaques and hyperphosphorylated tau. Postmortem studies have revealed that tau pathology is highly associated with the degree of cognitive impairment [1–3] and thus may serve as a biomarker of disease severity. In vivo tau can be captured in cerebrospinal fluid (CSF) and by positron emission tomography (PET). CSF phosphorylated tau (p-tau) is thought to reflect an AD-specific hyperphosphorylated state of tau [4]. [^18^F]flortaucipir is currently the most widely used PET tau tracer, which binds with high affinity to the paired helical filaments (PHFs) of tau in AD [5–7].

Although both are in vivo biomarkers for tau pathology, it remains unclear whether CSF p-tau and [^18^F]flortaucipir PET provide interchangeable or complementary information. So far, a number of studies have investigated associations between [^18^F]flortaucipir PET and CSF p-tau. Some studies found moderate to strong correlations across groups which consisted mainly of cognitively normal individuals [8, 9], while others could not replicate these findings [10]. Across the AD continuum, strongest associations between tau PET and CSF p-tau were mostly observed in later, dementia stages [11]. In addition, similar high levels were observed in CSF p-tau in prodromal AD and AD dementia, while [^18^F]flortaucipir uptake values continued to increase with progression of AD [12]. Taken together these results indicate that the relationship between CSF p-tau biomarkers and [^18^F]flortaucipir may depend on disease severity.

Both CSF p-tau [13, 14] and [^18^F]flortaucipir [15–21] have been related separately to proxies of disease severity, for example, to worse cognitive performance [13, 15–19] and a greater degree of atrophy [14, 18–21]. Despite these similarities, they may still differ on various aspects. [^18^F]flortaucipir PET is able to capture regional uptake patterns and mirrors established neuropathological staging schemes of tau [15, 22], while correlations of CSF p-tau with neuropathological tau burden have been modest [23, 24]. However, CSF p-tau may be more sensitive in detecting early changes in AD (tau) pathology [11, 25, 26]. Few studies have described the relationship between both CSF p-tau and [^18^F]flortaucipir binding with proxies of disease severity, and it remains to be established whether [^18^F]flortaucipir PET and p-tau can be viewed as equivalent markers for AD staging.

Therefore, we aimed to compare CSF and PET markers of tau pathology and to assess their relationship with cognitive impairment and atrophy as proxies of disease severity. We first examined regional associations between CSF p-tau and quantitative [^18^F]flortaucipir binding in cognitively normal and impaired subjects on the AD pathophysiological continuum [27, 28]. Second, to investigate whether CSF p-tau biomarkers and [^18^F]flortaucipir PET provide complementary information regarding disease severity, we investigated associations of both CSF p-tau and [^18^F]flortaucipir PET with multiple cognitive domains and regional atrophy.

## Methods

### Participants

We included 78 subjects from the Amsterdam Dementia Cohort [29, 30], of whom 25 were cognitively normal with subjective cognitive decline (SCD), and 53 were cognitively impaired (mild cognitive impairment (MCI) due to AD [31] (*n* = 8) or probable AD dementia [32] (*n* = 45)).

We grouped the MCI-AD and probable AD dementia subjects into one MCI/AD group.

All subjects underwent a standardized dementia screening, including medical history, extensive neuropsychological assessment, physical and neurological examination, lumbar puncture, blood tests, electroencephalography, and brain magnetic resonance imaging (MRI) [30]. Diagnosis was established by consensus in a multidisciplinary meeting [29, 30].

The label of SCD [33], which were used as controls, was provided based on self-reported cognitive complaints but without any objective impairment in performance on cognitive or neurological tasks or brain damage as evidenced by MRI. SCD subjects with evidence of substantial Aß pathology after visual reading of SUVR_50–70_ of [^18^F]florbetapir Aß-PET scans [34] were classified as amyloid positive subjects.

The diagnosis of MCI/AD met core clinical criteria [31, 32] according to the National Institute on Aging and Alzheimer’s Association (NIA-AA) and were biomarker supported, an AD like CSF (i.e., tau/Aβ42 fraction > 0.52 [35]) and/or a positive Aß-PET ([^11^C]PiB or [^18^F]florbetaben) scan by visual assessment [36, 37].

Exclusion criteria for all participants were (1) diagnosis of dementia not due to AD, (2) significant cerebrovascular disease on MRI (e.g., major CVA), (3) major traumatic brain injury, (4) major psychiatric or neurological disorders other than AD, (5) recent substance abuse, and (6) a time lag between LP and tau-PET imaging > 2 years. The study protocol was approved by the Medical Ethics Review Committee of the Amsterdam UMC, location VU Medical center. All patients provided written informed consent.

### CSF biomarkers

CSF samples were obtained from LP between the L3/L4, L4/L5, or L5/S1 intervertebral space and collected in polypropylene tubes using a 25-gauge needle and syringe [29]. To assess CSF biomarker levels, analyses were performed at the Neurochemistry Laboratory of the Department of Clinical Chemistry of the Amsterdam UMC according to international guidelines [38]. Levels of Aß_1–42_, t-tau, and p-tau (phosphorylated at threonine 181) in CSF were determined using sandwich ELISAs (Innotest *ß*-AMYLOID (1–42), Innotest hTAU-Ag, and Innotest PhosphoTAU-181p, Fujirebio (formerly Innogenetics), Belgium), and Aβ1–42 levels were corrected for drift in kit performance over time [39]. Cutoff values for abnormal CSF (Aβ1–42 < 813 pg/mL [39]), t-tau (> 375 pg/mL), and p-tau (> 52 pg/mL) [40] were used to define an AD like positive CSF profile (i.e., tau/Aβ42 fraction > 0.52 [35]) supportive of the diagnosis of MCI/AD. In this sample, p-tau and t-tau were highly correlated (*r* = 0.98, *p* < 0.01); therefore, all main analyses were performed with p-tau, and results for t-tau are provided in the Supplementary Material.

### Neuropsychological examination

The Mini-Mental State Examination (MMSE) was used as a measure of global cognitive status [41]. In addition, we assessed four cognitive domains [42] including memory (Dutch version of the Rey Auditory Verbal Learning Test immediate recall and delayed recall, Visual Association Test (VAT) version A), attention (Digit span Forward, Trial Making Test [TMT] version A), Stroop word and color naming), executive functioning (Digit span Backward, TMT version B, letter fluency test (D-A-T) and Stroop color-word interference test), and language (VAT-A naming and category fluency version animals). Trail Making Tests and Stroop tests were inverted so that lower scores indicated worse performance. For cognitive domain scores, we created composite scores by averaging Z-scores for each individual test within a domain (with a minimum of two tests per domain). The percentages of missing data for the cognitive domains were 10.3% (attention), 11.5% (memory), 12.8% (language), and 19.2% (executive functioning). As a sensitivity analysis we repeated our analyses including subjects without missing data (*n* = 59, see Supplementary Table 1).

### MRI imaging and processing

All subjects underwent a 3D-T1 weighted sequence on a 3.0 Tesla MRI scanner (Ingenuity TF PET/MR, Philips Medical Systems, Best, The Netherlands, *n* = 75), Vantage Titan (Toshiba Medical Systems, Otawara, Japan, *n* = 2), and a Signa HDxT (GE Healthcare, Milwaukee, WI, *n* = 1). All MR images were performed within a maximum of 12 months from the [^18^F]flortaucipir PET scan for SCD subjects (median = 0.2, IQR = 1.3) and a maximum of 6 months from the [^18^F]flortaucipir PET scan for subjects with MCI/AD (median = 0.1, IQR = 0.2).

We calculated gray matter (GM) volumes using voxel-based morphometry (VBM) implemented in Statistical Parametric Mapping (SPM) version 12 software (Wellcome Trust Center for Neuroimaging, University College London, UK) as described previously [43]. Structural T1-weighted MR images were segmented into gray matter, white matter, and cerebrospinal fluid. Quality control was performed on all gray matter native space images. Next, images were normalized to Montreal Neurological Institute (MNI) 152 space, using a study specific template created with the DARTEL toolbox. Whole brain gray matter maps were smoothed with an 8 mm full-width at half-maximum Gaussian kernel. We extracted regional gray matter volumes from the automatic anatomic labeling (AAL) using a priori defined cortical ROIs covering all the major brain lobes [43]: medial/lateral temporal, medial/lateral parietal, and occipital and frontal lobes (see Supplementary Table 2) based on the AAL atlas.

Total intracranial volume (TIV) was calculated by combining the native space segmentations (gray matter, white matter, and CSF) for each subject. We used gray matter density (i.e., gray matter volume corrected for TIV) as a proxy of atrophy in the analyses.

### [^18^F]flortaucipir imaging and processing

Dynamic 130 min [^18^F]flortaucipir PET scans were acquired on a Philips Ingenuity TF-64 PET/CT scanner. The scanning protocol consisted of two dynamic PET scans of 60 and 50 min, respectively, with a 20-min break in between [44, 45]. The first 60 min dynamic scan started simultaneously with a bolus injection 234 ± 16 MBq [^18^F]flortaucipir (injected mass 1.17 ± 0.78 μg). The second PET scan was co-registered to the first dynamic PET scan using Vinci software [46]. PET list mode data were rebinned into a total of 29 frames (1 × 15, 3 × 5, 3 × 10, 4 × 60, 2 × 150, 2 × 300, 4 × 600, and 10 × 300 seconds) and reconstructed using 3D RAMLA with a matrix size of 128 × 128 × 90 and a final voxel size of 2x2x2 mm^3^, including standard corrections for dead time, decay, attenuation, random, and scatter. The 3D-T1 MR images were co-registered to the averaged images (frame 8–29) of the PET scan.

The T1-weighted images were co-registered to their corresponding PET images using Vinci (volume imaging in neurological imaging) software [46]. Volumes of interest (VOI, Hammers template [47]) were subsequently delineated on the MR images and superimposed on the PET scan using PVElab [48]. Binding potential (BP_ND_) was generated using receptor parametric mapping (RPM) with whole cerebellar gray matter as a reference region (extracted from the Hammers template [47]). SUVr images were generated for the time interval 80–100 min post injection. BP_ND_ (or SUVr) images were resliced to FreeSurfer coordinates and using a MarsBar package in SPM12, BP_ND_ (or SUVr) was extracted in the following a priori defined FreeSurfer regions-of-interest (ROIs) on spatially normalized MR images: entorhinal, limbic, and neocortical regions. These ROIs closely aligning the neuropathological tau staging schemes, adjusted from Scholl et al. [15] (see Supplementary Table 3).

### Statistical analysis

Statistical analyses were performed using Statistical Package for the Social Sciences (SPSS, IBM version 22) and R (v. 3.2.3, The R foundation for Statistical computing).

Differences in demographic, clinical, and AD biomarker characteristics between disease groups were assessed using analyses of variance for continuous variables and *χ*^2^ for dichotomous data.

Linear regression models were performed to assess the relationship between entorhinal, limbic, and neocortical [^18^F]flortaucipir BP_ND_ (as dependent variable) and CSF p-tau (as independent variable), adjusted for age, sex, and time lag between LP and tau PET. We performed the analyses across all subjects and additionally stratified for diagnosis (i.e., SCD subjects and MCI/AD) and amyloid status for SCD subjects only. We did not stratify for amyloid status for the rest of the analyses because of the limited amount of amyloid positive SCD subjects (*n*  = 10).

In order to investigate spatial associations between CSF p-tau (independent variable) and [^18^F]flortaucipir BP_ND_ (or SUVr) (dependent variable), we performed a voxel-wise multiple regression analysis for CSF p-tau and [^18^F]flortaucipir PET_,_ adjusted for age, sex, and time lag between LP and [^18^F]flortaucipir PET scan. All resulting t-maps were thresholded with *p* < 0.001 at the voxel-level. Data are shown with and without correction for family-wise error *p* < 0.05.

Linear regression models were performed to assess the relationships between CSF p-tau and 3 a priori defined [^18^F]flortaucipir ROIs on PET with cognition. First, we assessed the predictive effects of CSF p-tau and entorhinal, limbic, and neocortical [^18^F]flortaucipir separately (model 1), followed by a model including both predictors (model 2: CSF p-tau + neocortical [^18^F]flortaucipir or entorhinal/limbic/neocortical [^18^F]flortaucipir + CSF p-tau as predictors). Additionally, both models were adjusted for age, sex, education, and time lag between PET/CSF assessments. Finally, we repeated the aforementioned procedure, but replaced cognition with gray matter atrophy (dependent variable) additionally adjusted for TIV.

In addition, all regressions were repeated by replacing BP_ND_ with SUVr_80-100min_ and were repeated by replacing CSF p-tau by t-tau. The results for t-tau are presented in the Supplementary Material. Results were  comparable for CSF p-tau and t-tau for all analysis.

For all linear regressions, standardized beta’s (sβ) were used as an outcome variable. By standardizing all variables in the equation, we obtain an easy interpretable outcome measure, which is comparable across regressions. The sβ is the change in the outcome variable for 1 standard deviation in change of the predictor. All sβ’s are tested against the null hypothesis that an sβ of 0 yields no effect [49].

## Results

### Demographics

Subject characteristics are presented in Table 1. Subjects were 64.8 ± 6.5 years old and as expected MCI/AD participant had a lower MMSE score of 23 ± 4 (*p* < 0.01). In addition, CSF p-tau and [^18^F]flortaucipir BP_ND_ within all brain regions were higher in MCI/AD patients compared to SCD subjects (all *p* < 0.01).

**Table 1:** Demographic, clinical, and AD biomarker characteristics over the total sample and per disease group

	SCD Aβ + (*n*=10)	SCD Aβ-(*n*=15)	MCI/AD (*n* = 53)	Total Sample (*n* = 78)	Total SCD (*n* = 25)
Age, years	67 ± 6	64 ± 6	65 ± 7	65 ± 7	65 ± 6
Female, %	60%	60%	53%	43%	60%
No. Aß positive subjects	10(100%)	0(0%)	53 (100%)	63 (81%)	10 (40%)
Education, Verhage scale, median(range)	6 (4-7)	6 (2-7)	6 (3-7)	6 (2-7)	6 (2-7)
Time lag LP/PET, years	0.7 ± 0.6	0.9 ± 0.5	0.6 ± 0.5	0.7 ± 0.5	0.8 ± 0.7
Neuropsychological measures					
MMSE (*n*=78)	28 ± 1	28 ± 1	23 ± 4^b^	25 ± 4	28 ± 1
Memory^*c*^ (*n*=78)	-0.5 ± 0.8	0.3 ± 0.6	-3.1 ± 2.1^b^	-2.1 ± 2.3	-0.0 ± 0.8
Attention^*d*^ (*n*=73)	-0.2 ± 0.6	0.1 ± 0.6	-1.3 ± 1.2^b^	-0.9 ± 1.2	-0.0 ± 0.6
Language^*e*^ (*n*=68)	-0.0 ± 0.4	0.0 ± 0.8	-1.0 ± 1.0^b^	-0.6 ± 1.0	-0.0 ± 0.7
Executive functioning^*f*^ (*n*=73)	-0.1 ± 0.9	-0.1 ± 0.7	-2.4 ± 1.0^b^	-0.9 ± 1.1	0.0 ± 0.8
Tau biomarkers					
CSF					
CSF Aß_1-42_	779 ± 197	1067 ± 217	541 ± 113^b^	677 ± 260	966 ± 247
CSF t-tau	615 ± 383	257 ± 201	760 ± 412^bd^	645 ± 422	401 ± 333
CSF p-tau	83 ± 36	43 ± 23	90 ± 35^b^	80 ± 38	59 ± 38
[^18^F]flortaucipir PET					
Entorhinal cortex BP_ND_	0.2 ± 0.2	-0.1 ± 0.1	0.3 ± 0.2^b^	0.2 ± 0.2	-0.0 ± 0.2
Limbic region BP_ND_	0.2 ± 0.1	0.0 ± 0.0	0.4 ± 0.2^b^	0.3 ± 0.2	0.1 ± 0.1
Neocortex BP_ND_	0.1 ± 0.1	-0.0 ± 0.0	0.3 ± 0.3^b^	0.2 ± 0.3	0.0 ± 0.1
Entorhinal cortex SUVr	1.0 ± 0.1	1.3 ± 0.2	1.5 ± 0.2^b^	1.4 ± 0.3	1.1 ± 0.2
Limbic region SUVr	1.1 ± 0.1	1.3 ± 0.2	1.5 ± 0.2^b^	0.4 ± 0.3	1.2 ± 0.1
Neocortex SUVr	1.1 ± 0.1	1.2 ± 0.2	1.4 ± 0.3^b^	1.3 ± 0.3	1. ± 0.1

Continuous data shown as mean ± standard deviation, unless specified otherwise. Differences in demographic, clinical, and AD biomarker characteristics between disease groups were assessed using ANOVA for continuous variables and *χ*^2^ for dichotomous data

^a^Significantly different from SCD subjects at *p* < 0.05. ^b^Significantly different from SCD subjects at *p* < 0.01

^c^Z-score memory domain, ^d^Z-score attention domain, ^e^Z-score language domain, ^f^Z-score executive functioning domain

### The relationship between CSF p-tau and [^18^F]flortaucipir BP_ND_

**Fig. 1:**
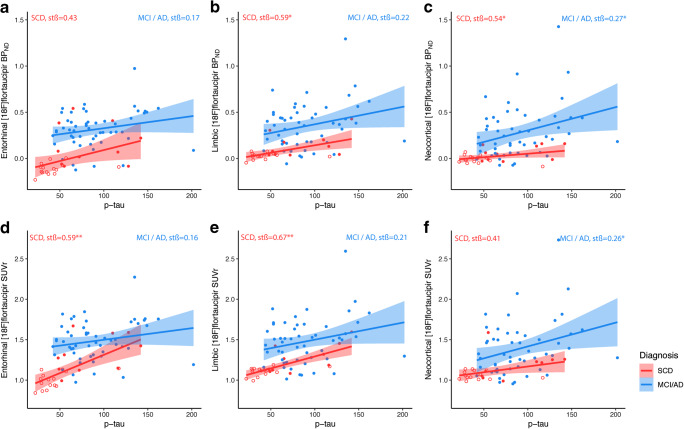
Scatterplots of the observed relationship between CSF p-tau with entorhinal, limbic, and neocortical [^18^F]flortaucipir BP_ND_ (top row, **a**–**c**) and SUVr (bottom row, **d**–**f**). Each symbol represents one subject. The fitted lines are stratified over AD (blue) and SCD subjects (red); closed circles are Aβ positive, open circles are Aβ negative. Correlations were adjusted for age, sex, and time lag between LP and [^18^F]flortaucipir PET scan

Associations between CSF p-tau and tau PET are presented in Table 2. Using all subjects CSF p-tau was associated with higher [^18^F]flortaucipir BP_ND_ in the entorhinal cortex (sβ = 0.46), limbic (sβ = 0.45), and neocortical region (sβ = 0.43), all *p* < 0.01. Within-group correlations were stronger for SCD subjects than for MCI/AD patients, with strongest correlations seen in the limbic region (sβ = 0.59, p < 0.01, Table 2, Fig. 1). Within the SCD subjects, the relationship between CSF p-tau and [^18^F]flortaucipir BP_ND_ was driven by the amyloid positive individuals (Fig. 1). Note that the variance of the SCD subjects patients is smaller than for MCI/AD patients, therefore a more gradual slope is observed for association between CSF p-tau and limbic, neocortical tau, although sβs are stronger for SCD. Comparable results were seen for CSF t-tau (Supplementary Table 4, Supplementary Fig. 1).

**Table 2:** Standardized *ß* coefficients for the relationship between CSF p-tau and entorhinal, limbic, and neocortical [^18^F]flortaucipir BP_ND_ or SUVr over the total sample and stratified per disease group

	Total sample (*n* = 78)	SCD (*n* = 25)	MCI/AD (*n* = 53)
	CSF p-tau	CSF p-tau	CSF p-tau
Entorhinal[^18^F]flortaucipir BP_ND_	**0.46** (*p* <0.01)	0.43 (*p* = 0.07)	0.17(*p* = 0.17)
Limbic [^18^F]flortaucipir BP_ND_	**0.45** (*p* = <0.01)	**0.59**^**a**^ (*p* = 0.01)	0.22 (*p* = 0.08)
Neocortical [^18^F]flortaucipir BP_ND_	**0.43** (*p* = <0.01)	**0.54** (*p* = 0.02)	**0.27** (*p* = 0.03)
Entorhinal[^18^F]flortaucipir SUVr	**0.50** (*p* = <0.01)	**0.59** (*p*<0.01)	0.16 (*p* = 0.21)
Limbic [^18^F]flortaucipir SUVr	**0.47** (*p* = <0.01)	**0.67**(*p* = 0.00)	0.21 (*p* = 0.09)
Neocortical [^18^F]flortaucipir SUVr	**0.43** (*p* = <0.01)	0.41 (*p* = 0.08)	**0.26** (*p* = 0.03)

Standardized *ß* coefficients (significant in bold) from regression analysis with [^18^F]flortaucipir BP_ND_ or SUVr as the dependent variables and CSF p-tau as predictor.

Effects adjusted for age, sex, and time lag between LP and [^18^F]flortaucipir PET

**Fig. 2:**
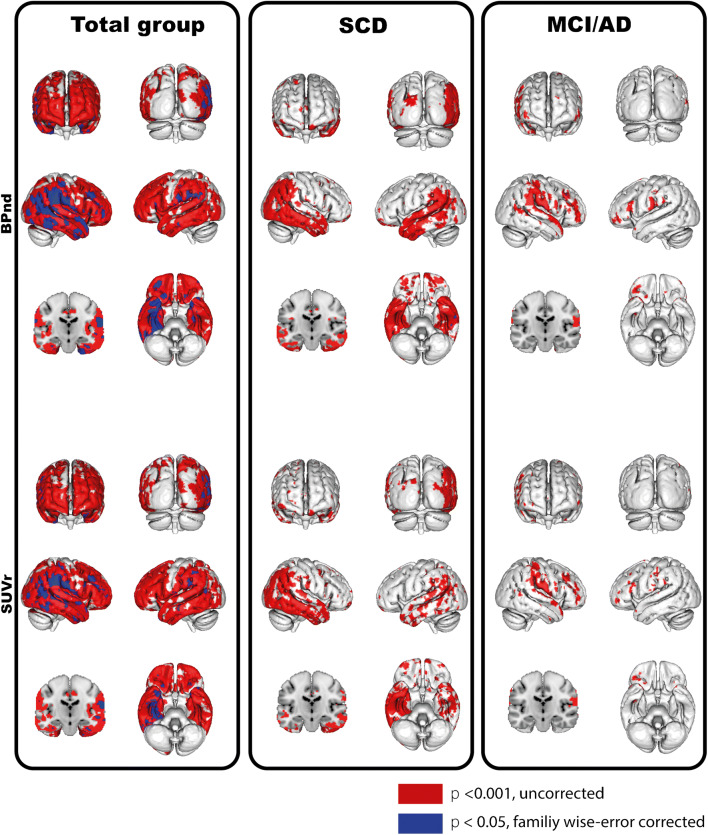
Voxel-wise associations between CSF p-tau and [^18^F]flortaucipir BP_ND_ (top row) and SUvr (bottom row). Voxel-wise associations are shown using a threshold *p*_uncorrected_ < 0.001(red) and *p*_FWEcorrected_ < 0.05 (blue) at the voxel level. Contrasts were adjusted for age, sex, and time lag between LP and [^18^F]flortaucipir PET scan. The associations were assessed in the total sample, within SCD subjects only and within MCI/AD subjects only

Voxel-wise analyses across all subjects showed significant associations between widespread cortical [^18^F]flortaucipir binding (right > left binding) and CSF p-tau. In SCD subjects, associations between tau PET and CSF p-tau were mainly observed in temporoparietal regions, whereas in MCI/AD this association was observed in the fronto-temporo-parietal areas (Fig. 2). All results survived family-wise error corrections, except for the results in the MCI/AD group. CSF t-tau showed a comparable, although marginally more widespread pattern than p-tau (Supplementary Fig. 2).

### Associations between CSF p-tau and [^18^F]flortaucipir BP_ND_ and cognition

Associations between CSF p-tau and tau PET and cognition are presented in Table 3 and Supplementary Table 1. Across the total group, all tau PET ROIs correlated to all cognitive scores: MMSE and the memory, attention and executive functioning, and language (model 1 with tau PET as a predictor; range sβ = − 0.23 to – 0.64, all *p* < 0.05). Contrary, CSF p-tau showed only associations with MMSE and memory (model 1; range sβ’s = − 0.26 to – 0.44, all *p* < 0.01, Table 3).

**Table 3:** Standardized *ß* coefficients for the relationship between cognitive outcome and CSF p-tau or entorhinal, limbic, and neocortical [^18^F]flortaucipir BP_ND_ over the total sample and stratified per disease group

	Total sample (*n* = 78)		SCD (*n* = 25)		MCI/AD (*n* = 53)	
	*Model 1*	*Model 2*	*Model 1*	*Model 2*	*Model 1*	*Model 2*
CSF p-tau						
MMSE	**− 0.44**^**b**^	**− 0.21**^**a**^	0.07	0.20	**− 0.29**^**a**^	− 0.17
Memory	**− 0.26**^**a**^	− 0.12	− 0.10	0.10	− 0.05	− 0.04
Attention	− 0.18	− 0.01	− 0.16	0.08	− 0.05	0.03
Executive functioning	− 0.14	0.06	0.28	**0.48**^**a**^	− 0.03	0.07
Language	− 0.10	− 0.00	0.11	0.32	− 0.00	0.01
[^18^F]flortaucipir BP_ND_						
Entorhinal region						
MMSE	**− 0.41**^**b**^	**− 0.25**^**a**^	− 0.23	− 0.21	− 0.01	− 0.04
Memory	**− 0.54**^**b**^	**− 0.52**^**b**^	**− 0.50**^**b**^	**− 0.50**^**a**^	− 0.27	− 0.27
Attention	− 0.14	− 0.35	− 0.17	− 0.14	**0.43**^**b**^	**0.45**^**b**^
Executive functioning	**− 0.29**^**b**^	0.25^a^	0.07	− 0.01	0.13	0.14
Language	**− 0.23**^**a**^	− 0.22	− 0.18	− 0.41	0.20	0.21
Limbic region						
MMSE	**− 0.64**^**b**^	**− 0.54**^**b**^	− 0.22	− 0.20	**− 0.50**^**b**^	**− 0.45**^**b**^
Memory	**− 0.46**^**b**^	**− 0.41**^**b**^	− 0.24	− 0.22	− 0.10	− 0.09
Attention	**− 0.45**^**b**^	**− 0.41**^**b**^	− 0.29	− 0.32	− 0.22	− 0.21
Executive functioning	**− 0.52**^**b**^	**− 0.52**^**b**^	− 0.06	− 0.23	**− 0.34**^**a**^	**− 0.34**^**a**^
Language	**− 0.33**^**b**^	**− 0.33**^**a**^	− 0.10	− 0.24	− 0.11	− 0.11
Neocortical region						
MMSE	**− 0.64**^**b**^	**− 0.53**^**b**^	− 0.17	− 0.13	**− 0.50**^**b**^	**− 0.43**^**b**^
Memory	**− 0.38**^**b**^	**− 0.29**^**a**^	− 0.32	− 0.29	0.04	0.07
Attention	**− 0.55**^**b**^	**− 0.52**^**b**^	**− 0.40**^**a**^	− 0.42	**− 0.38**^**a**^	**− 0.39**^**a**^
Executive functioning	**− 0.56**^**b**^	**− 0.57**^**b**^	− 0.16	− 0.31	**− 0.39**^**a**^	**− 0.41**^**a**^
Language	**− 0.27**^**a**^	− 0.25	− 0.25	− 0.39	0.00	0.02

Standardized *ß* coefficients (significant in bold) from multiple regression analysis with cognitive measures as the dependent variables and either CSF p-tau and/or [^18^F]flortaucipir BP_ND_ as predictors using separate analyses

Model 1 = Either CSF p-tau or entorhinal/limbic/neocortical [^18^F]flortaucipir BP_ND_ was used as a predictor. Effects adjusted for age, sex, education, and time lag between cognitive testing and LP or [^18^F]flortaucipir PET

Model 2 = CSF p-tau + neocortical [^18^F]flortaucipir BP_ND_ or entorhinal/limbic/neocortical [^18^F]flortaucipir BP_ND_ + CSF p-tau were used as predictors. Effects adjusted as model 1

^a^Significant standardized *ß* coefficient at *p* < 0.05

^b^Significant standardized *ß* coefficient at *p* < 0.01

When entered simultaneously (model 2), associations between [^18^F]flortaucipir BP_ND_ and cognition (model 2, MMSE, memory, attention, executive functioning, and language; range sβ’s = − 0.25 to – 0.57, all *p* < 0.05, Table 3) remained essentially comparable, but appeared strongly attenuated for CSF p-tau (model 2, MMSE; sβ = − 0.21, *p* < 0.05, Table 3).

After stratification, stronger associations were seen in MCI/ AD patients compared to SCD subjects. After correcting for the other biomarker (model 2), regional tau PET, but not CSF p-tau, was related to MMSE (range sβ’s = − 0.43 to – 0.45, all *p* < 0.01), attention (range sβ’s = − 0.39 to – 0.45, all *p* < 0.05), and executive functioning (range sβ’s = − 0.34 to – 0.41, all *p* < 0.05) within the MCI/AD patients. Comparable results for model 1 and model 2 were seen when replacing p-tau with t-tau (Supplementary Table 5).

### Associations between CSF p-tau and [^18^F]flortaucipir BP_ND_ and atrophy

Associations between CSF p-tau and tau PET and atrophy are presented in Table 4. Across all subjects, associations were seen between high CSF p-tau with temporal, parietal, and occipital atrophy (model 1; range sβ’s = − 0.20 to – 0.22, all *p* < 0.05), but disappeared when adjusting for neocortical tau PET. All [^18^F]flortaucipir ROIs were related to the different atrophy ROIs (model 1; range sβ’s = − 0.27 to – 0.49, all *p* < 0.05), even after adjusting for CSF p-tau (model 2; range sβ’s = − 0.20 to – 0.51, all *p* < 0.05). After stratification, these results appeared largely attributable to MCI/AD participants, no significant associations were seen for the SCD subjects. Comparable results for t-tau are presented in Supplementary Table 6.

**Table 4:** Standardized *ß* coefficients for the relationship between regional gray matter atrophy and CSF p-tau or [^18^F]flortaucipir BP_ND_ over the total sample and stratified per disease group

	Total sample (*n* = 78)		SCD (*n* = 25)		MCI/AD (*n* = 53)	
	*Model 1*	*Model 2*	*Model 1*	*Model 2*	*Model 1*	*Model 2*
CSF p-tau						
Medial temporal	− 0.15	− 0.01	0.18	0.25	0.03	0.08
Lateral temporal	**− 0.21**^**a**^	− 0.00	0.15	− 0.01	− 0.13	− 0.01
Medial parietal	− 0.17	0.04	0.17	0.29	− 0.09	0.04
Lateral parietal	**− 0.20**^**a**^	0.02	0.25	0.33	− 0.13	− 0.00
Frontal	− 0.10	0.09	0.21	0.33	− 0.07	0.04
Occipital	**− 0.22**^**a**^	− 0.15	0.15	0.30	− 0.18	− 0.07
[^18^F]flortaucipir BP_ND_						
Entorhinal region						
Medial temporal	**− 0.42**^**b**^	**− 0.45**^**b**^	− 0.14	− 0.22	**− 0.26**^**a**^	**− 0.27**^**a**^
Lateral temporal	**− 0.33**^**b**^	**− 0.31**^**b**^	− 0.09	− 0.16	− 0.23	− 0.20
Medial parietal	**− 0.27**^**b**^	**− 0.27**^**b**^	− 0.08	− 0.16	0.18	− 0.15
Lateral parietal	**− 0.31**^**b**^	**− 0.29**^**b**^	− 0.10	− 0.21	− 0.16	− 0.12
Frontal	**− 0.23**^**a**^	**− 0.25**^**a**^	− 0.19	− 0.32	− 0.11	− 0.09
Occipital	**− 0.27**^**b**^	− **0.20**^**a**^	− 0.21	− 0.32	− 0.14	− 0.09
Limbic region						
Medial temporal	**− 0.38**^**b**^	**− 0.40**^**b**^	− 0.14	− 0.20	**− 0.26**^**a**^	− 0.23
Lateral temporal	**− 0.48**^**b**^	**− 0.46**^**b**^	− 0.07	− 0.18	**− 0.50**^**b**^	**− 0.49**^**b**^
Medial parietal	**− 0.41**^**b**^	**− 0.43**^**b**^	− 0.11	− 0.26	**− 0.41**^**b**^	**− 0.40**^**b**^
Lateral parietal	**− 0.40**^**b**^	**− 0.41**^**b**^	− 0.01	− 0.26	**− 0.37**^**b**^	**− 0.35**^**b**^
Frontal	**− 0.31**^**b**^	**− 0.34**^**b**^	− 0.08	− 0.26	**− 0.29**^**a**^	**− 0.28**^**a**^
Occipital	**− 0.38**^**b**^	**− 0.37**^**b**^	− 0.16	− 0.32	**− 0.37**^**b**^	**− 0.34**^**b**^
Neocortical region						
Medial temporal	**− 0.32**^**b**^	**− 0.33**^**b**^	− 0.01	− 0.08	− 0.18	− 0.20
Lateral temporal	**− 0.47**^**b**^	**− 0.48**^**b**^	− 0.06	− 0.13	**− 0.48**^**b**^	**− 0.47**^**b**^
Medial parietal	**− 0.45**^**b**^	**− 0.48**^**b**^	− 0.09	− 0.18	**− 0.49**^**b**^	**− 0.49**^**b**^
Lateral parietal	**− 0.49**^**b**^	**− 0.51** ^**b**^	− 0.00	− 0.12	**− 0.50**^**b**^	**− 0.49**^**b**^
Frontal	**− 0.39**^**b**^	**− 0.44**^**b**^	− 0.11	− 0.24	**− 0.41**^**b**^	**− 0.40**^**b**^
Occipital	**− 0.47**^**b**^	**− 0.47**^**b**^	− 0.18	− 0.28	**− 0.50**^**b**^	**− 0.42**^**b**^

Standardized *ß* coefficients (significant in bold) from multiple regression analysis with gray matter density as the dependent variable and either CSF p-tau and/or [^18^F]flortaucipir BP_ND_ as predictors using separate analyses

Model 1 = Either CSF p-tau or entorhinal/limbic/neocortical [^18^F]flortaucipir BP_ND_ was used as a predictor. Effects adjusted for age, sex, intracranial volume, and time lag between MRI and LP or [^18^F]flortaucipir PET

Model 2 = CSF p-tau + neocortical [^18^F]flortaucipir BP_ND_ or entorhinal/limbic/neocortical [^18^F]flortaucipir BP_ND_ + CSF p-tau were used as predictors. Effects adjusted as model 1

^a^Significant standardized *ß* coefficient at *p* < 0.05

^b^Significant standardized *ß* coefficient at *p* < 0.01

### SUVr vs BP_ND_

Although SUVr overestimated BP_ND_ values (Table 1), comparable results were obtained for SUVr and BP_ND_. Overall, higher p-tau was related to higher entorhinal (sβ = 0.50), limbic (sβ = 0.47), and neocortical (sβ = 0.43) [^18^F]flortaucipir SUVr, all *p* < 0.01 (Table 2). In line with BP_ND_, within groups, correlations were stronger for SCD subjects, and strongest correlations were seen in the limbic region (sβ = 0.67 *p* < 0.01, Table 2, Fig. 1). Voxel-wise analysis confirmed comparable associations between [^18^F]flortaucipir BP_ND_ or SUVr and CSF p-tau (Fig. 2), and the associations with cognition and atrophy were essentially the same for SUVr (Supplementary Tables 7, 8) and BP_ND_ (Tables 3 and 4).

## Discussion

In this study, we examined cross-sectional associations between CSF p-tau and quantitative [^18^F]flortaucipir PET and their associations with cognition and atrophy. First, we found that higher CSF p-tau was only moderately correlated to higher [^18^F]flortaucipir binding across the total group. Both tau markers were associated with disease severity, but we found stronger associations for [^18^F]flortaucipir compared to CSF p-tau in relation to cognition and atrophy, particularly for the MCI/AD group. Taken together, our findings suggest that tau PET may be a better biomarker for tracking Alzheimer’s disease severity.

Our main finding was that tau PET, independently of CSF p-tau, was related to proxies of disease severity in AD. We found associations between various [^18^F]flortaucipir ROIs, recapitulating the neuropathological tau stages, and attention, memory, language, and executive functioning as well as with brain atrophy. Associations were most pronounced for MCI/AD dementia patients, especially with regard to limbic and neocortical tau deposition and widespread latrophy, which suggest that later neuropathological tau stages measured by [^18^F]flortaucipir BP_ND_ are tightly related to neurodegeneration in clinical AD. In line with the previous studies [15, 22], we showed that tau PET aligns with the neuropathological tau stages by showing a temporoparietal pattern in the SCD subjects and greater involvement of frontal areas in AD subjects. Previous studies also showed stronger associations for frontal, temporal, and parietal tau PET with cognition [11, 50] and temporoparietal [^18^F]flortaucipir with atrophy [11] compared to CSF p-tau in AD, further supporting the use of especially tau PET to track disease severity. A possible explanation for these differences in associations could be that each tau biomarker reflects different aspects of tau pathology. CSF p-tau rises early in the disease course, possibly even before Aβ PET positivity [51]. Additionally, extracellular soluble forms of pathological tau (as can be measured using CSF) may precede the intracellular aggregated hyperphosphorylated form of tau (as measured by [^18^F]flortaucipir PET). Therefore, CSF may show greater disparity than tau PET with later markers of disease severity, such as atrophy and cognition [52, 53].

After the initial early rise of CSF p-tau, it may have limited longitudinal changes in prodromal / AD dementia stages [54–56]. However, [^18^F]flortaucipir binding continues to increase over time [57, 58] suggesting a better ability for [^18^F]flortaucipir PET to dynamically track neurodegeneration and/or (changes in) cognition [59, 60] on the clinical stage of the disease. This is in line with our study, in which we observed that regional [^18^F]flortaucipir PET values were higher for MCI/AD patients than for SCD subject with similar p-tau values (Fig. 1). These findings underscore that tau PET may be a more robust marker for disease severity in clinical AD than CSF p-tau, and that tau PET may serve as an important outcome variable for tracking disease severity, for instance, during treatment with disease-modifying or symptomatic drugs. The early plateauing of CSF p-tau in the prodromal phase of AD may also explain the lack of correlation of p-tau and tau PET in MCI/AD patients. Indeed CSF p-tau tends to solely increase in Aβ + cognitively normal individuals and patients with MCI and stabilize or even decrease in AD dementia [54]. Alternatively, results may be partly be explained by differences in age (65 years (present study) vs. 74–77 years) [10, 11]. Early onset AD harbors a greater tau pathology burden and faster progression than late onset AD [61, 62], and studies have shown that age could affect the levels of p-tau [63] and the amount of [^18^F]flortaucipir uptake [18, 64].

In line with earlier studies [8], we observed a tight link between CSF p-tau and [^18^F]flortaucipir BP_ND_ in SCD. However, other studies did not find a relationship between CSF p-tau and [^18^F]flortaucipir within cognitively unimpaired controls [9–11], which could be related to the notion that individuals with SCD are at increased risk for AD [65, 66, 67]. However, p-tau may be more sensitive in early stages than [^18^F]flortaucipir [11, 26, 52], as subtle increases in [^18^F]flortaucipir binding has been shown in the preclinical stages of AD [15, 19, 60, 68].

Additionally, by using quantitative [^18^F]flortaucipir BP_ND_, we may have been able to capture modest increases in [^18^F]flortaucipir uptake in SCD subjects, since we are able to measure small effects with BP_ND_ with higher test-rest accuracy than SUVr [69]. Although in this study, we observed comparable results if we replace BP_ND_ with SUVr. In line with the previous studies, we found that SUVr overestimated BP_ND_ [70]; however, this appeared to only marginally affect the associations of p-tau and tau PET.

### Strengths and limitations

The strength of this study is that we used the dynamic tau PET scans resulting in specific tracer to tau binding rather than a semi-quantitative approach (i.e., SUVr) which overestimates true tracer binding as shown in this study.

In addition, we examined these associations between the different tau biomarkers in SCD subjects instead of cognitively unimpaired individuals without cognitive complaints. By not using controls from a populations study, these results may be more generalizable to a memory clinic cohort.

We acknowledge that there was a time lag in our study between CSF tau sampling and performing of the tau PET scan. Ideally, CSF would be collected on the day of the PET scan, and although our results were comparable if we adjusted the time lag between CSF p-tau and tau PET to a maximum of 1 year and we entered the time lag as a covariate in our main analysis, this time lag may have affected our results. Since p-tau levels may rise before tau PET [11, 26, 52], associations may have been stronger when there was a greater time lag.

## Conclusion

Regional [^18^F]flortaucipir PET, more than CSF p-tau, relates to important clinical parameters of disease severity of AD, i.e., cognition and neurodegeneration. As such, tau PET may more accurately reflect disease severity in AD than p-tau.

## Electronic supplementary material

ESM 1 (DOCX 18 kb)

ESM 2 (DOCX 14 kb)

ESM 3 (DOCX 16 kb)

ESM 4 (DOCX 14 kb)

ESM 5 (DOCX 18 kb)

ESM 6 (DOCX 18 kb)

ESM 7 (DOCX 18 kb)

ESM 8 (DOCX 18 kb)

ESM 9 Scatterplots of the observed relationship between CSF p-tau with entorhinal, limbic, and neocortical [^18^F]flortaucipir BP_ND_. Each symbol represents one subject. The fitted lines are stratified over AD (blue) and SCD subjects (red); closed circles are Aβ positive, open circles are Aβ negative. Correlations were adjusted for age, sex, and time lag between LP and [^18^F]flortaucipir PET scan. (PNG 12830 kb)

High resolution image (TIF 13503 kb)

ESM 10 Voxel-wise associations between CSF t-tau and [^18^F]flortaucipir BP_ND._ Voxel-wise associations are shown using a threshold *p*_uncorrected_ < 0.001(red) and *p*_FWEcorrected_ < 0.05 (blue) at the voxel level. Contrasts were adjusted for age, sex, and time lag between LP and [^18^F]flortaucipir PET scan. The associations were assessed in the total sample, within SCD subjects only and within MCI/AD subjects only. (PNG 2198 kb)

High resolution image (TIF 20022 kb)
