# Supplementary material for: Regional [18F]flortaucipir PET is more closely associated with disease severity than CSF p-tau in Alzheimer’s disease
Source: Eur J Nucl Med Mol Imaging. 2020 Apr 14;47(12):2866–78. doi: 10.1007/s00259-020-04758-2 (PMC7567681; doi:10.1007/s00259-020-04758-2)
Supplement: Supplementary file 1 — (DOCX 18 kb) [file 259_2020_4758_MOESM1_ESM.docx]

|  | Total Sample  (n=59) | | SCD  (n=21) | | MCI/AD  (n=38) | |
| --- | --- | --- | --- | --- | --- | --- |
|  | *Model 1* | *Model 2* | *Model 1* | *Model 2* | *Model 1* | *Model 2* |
| CSF  p-tau |  |  |  |  |  |  |
| MMSE | **-0.26^b^** | -0.02 | 0.09 | 0.26 | -0.11 | 0.07 |
| Memory | **-**0.22 | -0.06 | -0.12 | 0.08 | -0.07 | -0.05 |
| Attention | -0.13 | 0.08 | -0.18 | 0.13 | -0.01 | 0.14 |
| Executive functioning | -0.10 | 0.17 | 0.25 | **0.51^a^** | -0.04 | 0.18 |
| Language | -0.05 | 0.09 | 0.06 | 0.27 | 0.06 | 0.10 |
| [^18^F]flortaucipir BP_ND_  Entorhinal region |  |  |  |  |  |  |
| MMSE | **-0.53^b^** | **-0.52^b^** | **-0.49^b^** | **-0.72^b^** | -0.14 | -0.11 |
| Memory | **-0.62^b^** | **-0.65^b^** | **-0.56^a^** | **-0.70^a^** | -0.26 | -0.26 |
| Attention | **-0.25^a^** | -0.21 | -0.35 | -0.52 | 0.30 | 0.33 |
| Executive functioning | **-0.41^b^** | **-0.46^b^** | -0.16 | **-0.61^a^** | -0.01 | 0.01 |
| Language | **-0.49^a^** | -0.29 | -0.15 | -0.33 | 0.23 | 0.23 |
| Limbic region |  |  |  |  |  |  |
| MMSE | **-0.60^b^** | **-0.60^b^** | -0.39 | -0.50 | **-0.45^b^** | **-0.47^a^** |
| Memory | **-0.50^b^** | **-0.47^b^** | -0.31 | -0.22 | -0.16 | -0.15 |
| Attention | **-0.41^b^** | **-0.41^b^** | -0.40 | -0.55 | -0.20 | -0.22 |
| Executive functioning | **-0.52^b^** | **-0.58^b^** | -0.15 | **-0.48^a^** | **-0.40^a^** | **-0.44^a^** |
| Language | **-0.32^b^** | **-0.36^a^** | -0.12 | -0.23 | -0.09 | -0.12 |
| Neocortical region |  |  |  |  |  |  |
| MMSE | **-0.56^b^** | **-0.54^b^** | -0.31 | -0.33 | **-0.41^a^** | **-0.45^a^** |
| Memory | **-0.43^b^** | **-0.37^a^** | -0.43 | -0.38 | -0.06 | -0.02 |
| Attention | **-0.48^b^** | **-0.50^b^** | **-0.52^a^** | **-0.61^a^** | -0.37 | **-0.45^a^** |
| Executive functioning | **-0.52^b^** | **-0.60^b^** | -0.26 | **-0.48^a^** | **-0.41^a^** | **-0.50^b^** |
| Language | **-**0.26 | -0.28 | 0.28 | -0.40 | 0.02 | 0.02 |
| Supplementary Table 1 Standardized ß coefficients for the relationship between cognitive outcome and entorhinal, limbic and neocortical [^18^F]flortaucipir over the total sample and stratified per disease group for subjects with complete neuropsychological examination.  Standardized ß coefficients (significant in bold) from multiple regression analysis with cognitive measures as the dependent variables and either CSF p-tau and/ or [^18^F]flortaucipir BP_ND_ as predictors using separate analyses.  Model 1 = Either CSF p-tau or entorhinal/limbic/neocortical [^18^F]flortaucipir BP_ND_ was used as a predictor. Effects adjusted for age, sex, education, and time lag between cognitive testing and LP or [^18^F]flortaucipir PET  Model 2 = CSF p-tau + neocortical [^18^F]flortaucipir BP_ND_ or entorhinal/limbic/neocortical [^18^F]flortaucipir BP_ND_ + CSF p-tau were used as predictors. Effects adjusted as model 1.  ^a^ Significant standardized ß coefficient at p < 0.05  ^b^ Significant standardized ß coefficient at p < 0.01 | | | | | | |
