# Supplementary material for: Regional [18F]flortaucipir PET is more closely associated with disease severity than CSF p-tau in Alzheimer’s disease
Source: Eur J Nucl Med Mol Imaging. 2020 Apr 14;47(12):2866–78. doi: 10.1007/s00259-020-04758-2 (PMC7567681; doi:10.1007/s00259-020-04758-2)
Supplement: Supplementary file 2 — (DOCX 14 kb) [file 259_2020_4758_MOESM2_ESM.docx]

| **Table S2** Overview of included regions AAL regions for the regional brain lobes approach for atrophy | |
| --- | --- |
| **Regional approach**  **(Brain lobes)** | **AAL-derived ROI** |
| Medial temporal | Hippocampus, parahippocampal gyrus, amygdala |
| Lateral temporal | Inferior temporal gyrus, middle temporal gyrus, superior temporal gyrus |
| Medial parietal  Lateral parietal  Frontal  Occipital | Posterior cingulum, precuneus  Inferior parietal gyrus, superior parietal gyrus, supramarginal gyrus  Middle frontal gyrus, superior frontal gyrus  Inferior occipital gyrus, middle occipital gyrus, superior occipital gyrus |
|  | |

**Supplementary table 2** Overview of included AAL regions within the brain lobe ROIs
