# Supplementary material for: Regional [18F]flortaucipir PET is more closely associated with disease severity than CSF p-tau in Alzheimer’s disease
Source: Eur J Nucl Med Mol Imaging. 2020 Apr 14;47(12):2866–78. doi: 10.1007/s00259-020-04758-2 (PMC7567681; doi:10.1007/s00259-020-04758-2)
Supplement: Supplementary file 3 — (DOCX 16 kb) [file 259_2020_4758_MOESM3_ESM.docx]

| **Table S3** Overview of included regions FreeSurfer regions for the regional [^18^F]flortaucipir Braak & Braak approach | |
| --- | --- |
| **Regional approach**  **(similar to Braak & Braak)** | **FreeSurfer-derived ROI** |
| Stage I-II | Entorhinal cortex |
| Stage III-IV | Parahippocampal gyrus; fusiform gyrus; lingual gyrus; amygdala; inferior temporal cortex; middle temporal cortex; temporal pole; thalamus; caudal, rostral, isthmus, posterior cingulate; insula |
| Stage V-VI | Frontal cortex; parietal cortex; occipital cortex; transverse, superior temporal cortex; precuneus; banks of superior temporal sulcus; nucleus accumbens; caudate nucleus; putamen; precentral gyrus; postcentral gyrus; paracentral gyrus; cuneus; pericalcarine |
|  | |

**Supplementary table 3** Overview of included FreeSurfer regions within the [^18^F]flortaucipir ROIs similar to Braak & Braak staging, adjusted from Scholl et al. 2016[15]
