# Supplementary material for: Regional [18F]flortaucipir PET is more closely associated with disease severity than CSF p-tau in Alzheimer’s disease
Source: Eur J Nucl Med Mol Imaging. 2020 Apr 14;47(12):2866–78. doi: 10.1007/s00259-020-04758-2 (PMC7567681; doi:10.1007/s00259-020-04758-2)
Supplement: Supplementary file 4 — (DOCX 14 kb) [file 259_2020_4758_MOESM4_ESM.docx]

|  | Total Sample  (n=78) | SCD  (n=25) | MCI/AD  (n=53) |
| --- | --- | --- | --- |
|  | CSF t-tau | CSF t-tau | CSF t-tau |
| Entorhinal[^18^F]flortaucipir BP_ND_ | **0.48^b^** | 0.43 | 0.21 |
| Limbic [^18^F]flortaucipir BP_ND_ | **0.48^b^** | **0.71^b^** | **0.25^a^** |
| Neocortical [^18^F]flortaucipir BP_ND_ | **0.45^b^** | **0.59^b^** | 0.28 |

**Supplementary Table 4** Standardized ß coefficients for the relationship between CSF t-tau and entorhinal, limbic and neocortical[^18^F]flortaucipir BP_ND_ over the total sample and stratified per disease group.

Standardized ß coefficients (significant in bold) from regression analysis with [^18^F]flortaucipir BP_ND_ as the dependent variables and CSF t-tau as predictor .

Effects adjusted for age, sex and time lag between LP and [^18^F]flortaucipir PET

^a^ p < 0.05

^b^ p < 0.01
