# Supplementary material for: Regional [18F]flortaucipir PET is more closely associated with disease severity than CSF p-tau in Alzheimer’s disease
Source: Eur J Nucl Med Mol Imaging. 2020 Apr 14;47(12):2866–78. doi: 10.1007/s00259-020-04758-2 (PMC7567681; doi:10.1007/s00259-020-04758-2)
Supplement: Supplementary file 5 — (DOCX 18 kb) [file 259_2020_4758_MOESM5_ESM.docx]

|  | Total Sample  (n=78) | | SCD  (n=25) | | | MCI/AD  (n=53) | | |
| --- | --- | --- | --- | --- | --- | --- | --- | --- |
|  | *Model 1* | *Model 2* | | *Model 1* | *Model 2* | | *Model 1* | *Model 2* |
| CSF |  |  | |  |  | |  |  |
| T-tau |  |  | |  |  | |  |  |
| MMSE | **-0.49^b^** | **-0.27^a^** | | 0.01 | 0.15 | | **-0.35^a^** | -0.23 |
| Memory | **-0.29^b^** | -0.16 | | -0.09 | 0.27 | | -0.09 | -0.06 |
| Attention | -0.22 | -0.03 | | -0.24 | -0.00 | | -0.08 | 0.01 |
| Executive functioning | **-0.23^a^** | -0.02 | | 0.19 | 0.41 | | -0.13 | -0.03 |
| Language | -0.17 | -0.08 | | 0.07 | 0.31 | | -0.09 | -0.09 |
| [^18^F]flortaucipir BP_ND_  Entorhinal region |  |  | |  |  | |  |  |
| MMSE | **-0.41^b^** | -0.22 | | -0.23 | -0.19 | | -0.01 | 0.08 |
| Memory | **-0.54^b^** | **-0.51^b^** | | **-0.50^b^** | **-0.51^a^** | | -0.27 | -0.26 |
| Attention | -0.14 | -0.03 | | -0.17 | -0.11 | | **0.43^a^** | **0.46^b^** |
| Executive functioning | **-0.29^b^** | -0.22 | | 0.07 | 0.02 | | 0.13 | 0.16 |
| Language | **-0.23^a^** | -0.18 | | -0.18 | -0.37 | | 0.20 | 0.22 |
| Limbic region |  |  | |  |  | |  |  |
| MMSE | **-0.64^b^** | **-0.51^b^** | | -0.22 | -0.17 | | **-0.50^b^** | **-0.42^b^** |
| Memory | **-0.46^b^** | **-0.39^b^** | | -0.24 | -0.24 | | -0.10 | -0.08 |
| Attention | **-0.45^b^** | **-0.40^b^** | | -0.29 | -0.28 | | -0.22 | -0.21 |
| Executive functioning | **-0.52^b^** | **-0.50^b^** | | -0.06 | -0.23 | | **-0.34^a^** | **-0.32^a^** |
| Language | **-0.33^b^** | **-0.30^a^** | | -0.10 | -0.29 | | -0.11 | -0.09 |
| Neocortical region |  |  | |  |  | |  |  |
| MMSE | **-0.64^b^** | **-0.49^b^** | | -0.17 | -0.10 | | **-0.50^b^** | **-0.41^a^** |
| Memory | **-0.38^b^** | -0.27 | | -0.32 | -0.31 | | 0.04 | 0.08 |
| Attention | **-0.55^b^** | **-0.51^b^** | | **-0.40^a^** | -0.39 | | **-0.38^a^** | **-0.39^a^** |
| Executive functioning | **-0.56^b^** | **-0.54^b^** | | -0.16 | -0.30 | | **-0.39^a^** | **-0.38^a^** |
| Language | **-0.27^a^** | -0.22 | | -0.25 | -0.40 | | 0.00 | 0.05 |

**Supplementary Table 5** Standardized ß coefficients for the relationship between cognitive outcome and CSF t-tau or entorhinal, limbic and neocortical [^18^F]flortaucipir BP_ND_ over the total sample and stratified per disease group.

Standardized ß coefficients (significant in bold) from multiple regression analysis with cognitive measures as the dependent variables and either CSF t-tau and/ or [^18^F]flortaucipir BP_ND_ as predictors using separate analyses.

Model 1 = Either CSF t-tau or entorhinal/limbic/neocortical [^18^F]flortaucipir BP_ND_ was used as a predictor. Effects adjusted for age, sex, education, and time lag between cognitive testing and LP or [^18^F]flortaucipir PET

Model 2 = CSF t-tau + neocortical [^18^F]flortaucipir BP_ND_ or entorhinal/limbic/neocortical [^18^F]flortaucipir BP_ND_ + CSF t-tau were used as predictors. Effects adjusted as model 1**.**

^a^ Significant standardized ß coefficient at p < 0.05.

^b^ Significant standardized ß coefficient at p < 0.01.
