# Supplementary material for: Regional [18F]flortaucipir PET is more closely associated with disease severity than CSF p-tau in Alzheimer’s disease
Source: Eur J Nucl Med Mol Imaging. 2020 Apr 14;47(12):2866–78. doi: 10.1007/s00259-020-04758-2 (PMC7567681; doi:10.1007/s00259-020-04758-2)
Supplement: Supplementary file 6 — (DOCX 18 kb) [file 259_2020_4758_MOESM6_ESM.docx]

|  | Total Sample  (n=78) | | SCD  (n=25) | | MCI/AD  (n=53) | |
| --- | --- | --- | --- | --- | --- | --- |
|  | *Model 1* | *Model 2* | *Model 1* | *Model 2* | *Model 1* | *Model 2* |
| CSF |  |  |  |  |  |  |
| T-tau |  |  |  |  |  |  |
| Medial temporal | **-0.22^a^** | -0.09 | 0.10 | 0.16 | -0.20 | 0.02 |
| Lateral temporal | **-0.27^b^** | -0.07 | 0.07 | 0.16 | -0.19 | -0.07 |
| Medial parietal | **-0.21^a^** | 0.00 | 0.09 | 0.21 | -0.11 | 0.02 |
| Lateral parietal | **-0.24^a^** | -0.02 | 0.20 | 0.29 | -0.15 | -0.02 |
| Frontal | -0.15 | 0.03 | 0.15 | 0.29 | -0.10 | -0.01 |
| Occipital | **-0.26^b^** | -0.06 | 0.08 | 0.24 | -0.20 | -0.08 |
| [^18^F]flortaucipir BP_ND_ |  |  |  |  |  |  |
| Entorhinal region |  |  |  |  |  |  |
| Medial temporal | **-0.42^b^** | **-0.41^b^** | -0.14 | -0.19 | **-0.26^a^** | **-0.26^a^** |
| Lateral temporal | **-0.33^b^** | **-0.27^b^** | -0.09 | -0.13 | -0.23 | -0.18 |
| Medial parietal | **-0.27^b^** | **-0.24^a^** | -0.08 | -0.13 | 0.18 | -0.14 |
| Lateral parietal | **-0.31^b^** | **-0.26^a^** | -0.10 | -0.19 | -0.16 | -0.11 |
| Frontal | **-0.23^a^** | **-0.22^a^** | -0.19 | -0.29 | -0.11 | -0.08 |
| Occipital | **-0.27^b^** | -0.20 | -0.21 | -0.29 | -0.14 | -0.08 |
| Limbic region |  |  |  |  |  |  |
| Medial temporal | **-0.38^b^** | **-0.36^b^** | -0.14 | -0.09 | **-0.26^a^** | **-0.23^a^** |
| Lateral temporal | **-0.48^b^** | **-0.46^b^** | -0.07 | -0.18 | **-0.50^b^** | **-0.48^b^** |
| Medial parietal | **-0.41^b^** | **-0.41^b^** | -0.11 | -0.26 | **-0.41^b^** | **-0.39^b^** |
| Lateral parietal | **-0.40^b^** | **-0.38^b^** | -0.01 | -0.20 | **-0.37^b^** | **-0.34^b^** |
| Frontal | **-0.31^b^** | **-0.31^b^** | -0.08 | -0.30 | **-0.29^a^** | **-0.27^a^** |
| Occipital | **-0.38^b^** | **-0.35^b^** | -0.16 | -0.34 | **-0.37^b^** | **-0.32^b^** |
| Neocortical region |  |  |  |  |  |  |
| Medial temporal | **-0.32^b^** | **-0.29^b^** | -0.01 | -0.05 | -0.18 | -0.17 |
| Lateral temporal | **-0.47^b^** | **-0.44^b^** | -0.06 | -0.11 | **-0.48^b^** | **-0.44^b^** |
| Medial parietal | **-0.45^b^** | **-0.46^b^** | -0.09 | -0.16 | **-0.49^b^** | **-0.48^b^** |
| Lateral parietal | **-0.49^b^** | **-0.48^b^** | -0.00 | -0.11 | **-0.50^b^** | **-0.48^b^** |
| Frontal | **-0.39^b^** | **-0.41^b^** | -0.11 | -0.24 | **-0.41^b^** | **-0.40^b^** |
| Occipital | **-0.47^b^** | **-0.45^b^** | -0.18 | -0.27 | **-0.50^b^** | **-0.46^b^** |

**Supplementary Table 6** Standardized ß coefficients for the relationship between regional grey matter atrophy and CSF t-tau or [^18^F]flortaucipir BP_ND_ over the total sample and stratified per disease group.

Standardized ß coefficients (significant in bold) from multiple regression analysis with grey matter density as the dependent variable and CSF t-tau and/ or [^18^F]flortaucipir BP_ND_ as predictors using separate analyses.

Model 1 = Either CSF t-tau or entorhinal/limbic/neocortical [^18^F]flortaucipir BP_ND_ was used as a predictor. Effects adjusted for age, sex, intracranial volume and time lag between MRI and LP or [^18^F]flortaucipir PET

Model 2 = CSF t-tau + neocortical [^18^F]flortaucipir BP_ND_ or entorhinal/limbic/neocortical [^18^F]flortaucipir BP_ND_ + CSF t-tau were used as predictors. Effects adjusted as model 1**.**

^a^ Significant standardized ß coefficient at p < 0.05.

^b^ Significant standardized ß coefficient at p < 0.01.
