# Supplementary material for: Regional [18F]flortaucipir PET is more closely associated with disease severity than CSF p-tau in Alzheimer’s disease
Source: Eur J Nucl Med Mol Imaging. 2020 Apr 14;47(12):2866–78. doi: 10.1007/s00259-020-04758-2 (PMC7567681; doi:10.1007/s00259-020-04758-2)
Supplement: Supplementary file 7 — (DOCX 18 kb) [file 259_2020_4758_MOESM7_ESM.docx]

|  | Total Sample  (n=78) | | SCD  (n=25) | | | MCI/AD  (n=53) | | |
| --- | --- | --- | --- | --- | --- | --- | --- | --- |
|  | *Model 1* | *Model 2* | | *Model 1* | *Model 2* | | *Model 1* | *Model 2* |
| CSF  p-tau |  |  | |  |  | |  |  |
| MMSE | **-0.44^b^** | **-0.23^a^** | | 0.07 | 0.03 | | **-0.29^a^** | -0.16 |
| Memory | **-0.26^a^** | -0.14 | | -0.10 | 0.12 | | -0.05 | -0.03 |
| Attention | -0.18 | -0.04 | | -0.16 | 0.08 | | -0.05 | 0.03 |
| Executive functioning | -0.14 | 0.03 | | 0.28 | 0.24 | | -0.03 | 0.07 |
| Language | -0.10 | -0.05 | | 0.11 | 0.02 | | -0.00 | -0.00 |
| [^18^F]flortaucipir SUVr  Entorhinal region |  |  | |  |  | |  |  |
| MMSE | **-0.37^b^** | -0.19 | | -0.16 | -0.11 | | 0.01 | 0.07 |
| Memory | **-0.54^b^** | **-0.53^b^** | | -0.42 | -0.50 | | **-0.32^a^** | **-0.32^a^** |
| Attention | -0.07 | 0.05 | | 0.04 | 0.22 | | **0.45^b^** | **0.47^b^** |
| Executive functioning | **-0.22^a^** | 0.17 | | 0.37 | 0.40 | | 0.15 | 0.16 |
| Language | -0.13 | -0.09 | | 0.06 | 0.03 | | 0.26 | 0.26 |
| Limbic region |  |  | |  |  | |  |  |
| MMSE | **-0.63^b^** | **-0.53^b^** | | -0.13 | -0.07 | | **-0.51^b^** | **-0.46^b^** |
| Memory | **-0.46^b^** | **-0.41^b^** | | -0.12 | 0.04 | | -0.16 | -0.16 |
| Attention | **-0.41^b^** | **-0.36^b^** | | -0.06 | 0.05 | | -0.20 | -0. 19 |
| Executive functioning | **-0.47^b^** | **-0.47^b^** | | 0.26 | 0.23 | | **-0.33^a^** | **-0.33^a^** |
| Language | -0.24 | -0.21 | | 0.20 | 0.32 | | -0.05 | -0.04 |
| Neocortical region |  |  | |  |  | |  |  |
| MMSE | **-0.61^b^** | **-0.49^b^** | | -0.02 | 0.06 | | **-0.52^b^** | **-0.45^b^** |
| Memory | **-0.36^b^** | -0.26 | | -0.08 | 0.01 | | 0.04 | 0.02 |
| Attention | **-0.50^b^** | **-0.55^b^** | | -0.11 | -0.07 | | -0.37 | -0.37 |
| Executive functioning | **-0.50^b^** | **-0.49^b^** | | 0.17 | 0.11 | | **-0.37^a^** | **-0.40^a^** |
| Language | -0.15 | -0.11 | | 0.24 | 0.26 | | 0.06 | 0.08 |

**Supplementary table 7** Standardized ß coefficients for the relationship between cognitive outcome and CSF p-tau or entorhinal, limbic and neocortical [^18^F]flortaucipir SUVr over the total sample and stratified per disease group.

Standardized ß coefficients (significant in bold) from multiple regression analysis with cognitive measures as the dependent variables and either CSF p-tau and/ or [^18^F]flortaucipir SUVr as predictors using separate analyses.

Model 1 = Either CSF p-tau or entorhinal/limbic/neocortical [^18^F]flortaucipir SUVr was used as a predictor. Effects adjusted for age, sex, education, and time lag between cognitive testing and LP or [^18^F]flortaucipir PET

Model 2 = CSF p-tau + neocortical [^18^F]flortaucipir SUVr or entorhinal/limbic/neocortical [^18^F]flortaucipir SUVr + CSF t-tau were used as predictors. Effects adjusted as model 1**.**

^a^ Significant standardized ß coefficient at p < 0.05.

^b^ Significant standardized ß coefficient at p < 0.01.
