# Supplementary figures and images for: Regional [18F]flortaucipir PET is more closely associated with disease severity than CSF p-tau in Alzheimer’s disease
Source: Eur J Nucl Med Mol Imaging. 2020 Apr 14;47(12):2866–78. doi: 10.1007/s00259-020-04758-2 (PMC7567681; doi:10.1007/s00259-020-04758-2)

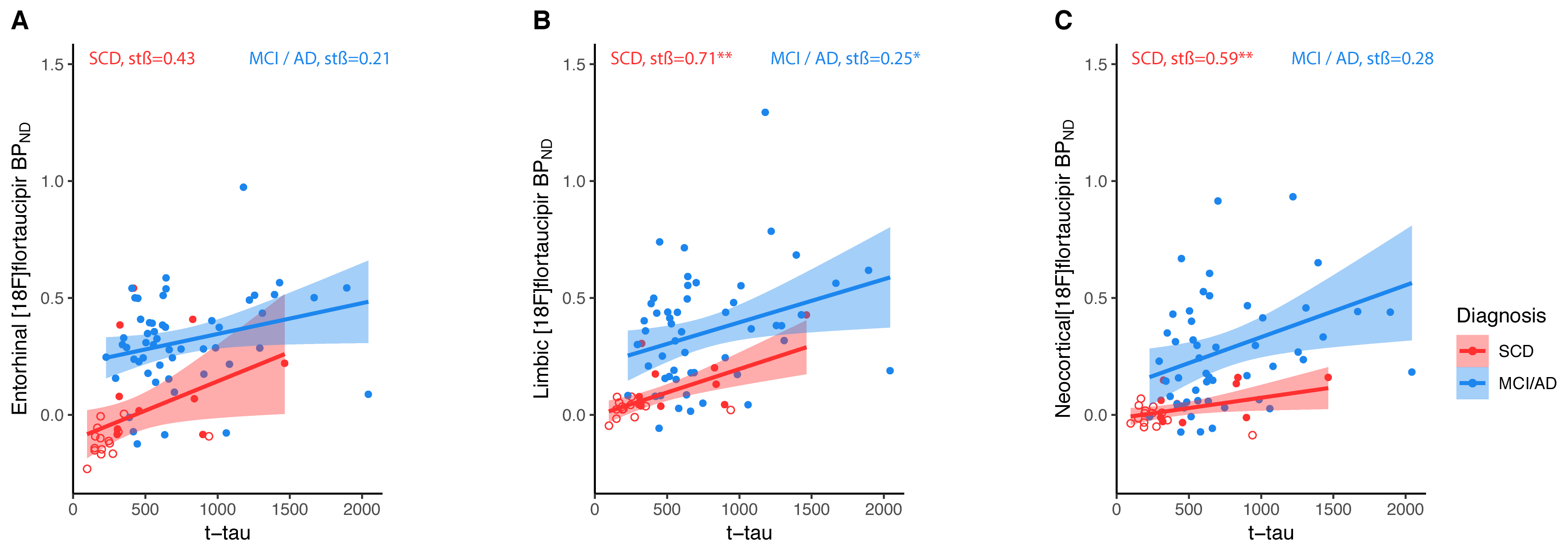

Supplement: Supplementary file 9 — Scatterplots of the observed relationship between CSF p-tau with entorhinal, limbic, and neocortical [18F]flortaucipir BPND. Each symbol represents one subject. The fitted lines are stratified over AD (blue) and SCD subjects (red); closed circles are Aβ positive, open circles are Aβ negative. Correlations were adjusted for age, sex, and time lag between LP and [18F]flortaucipir PET scan. (PNG 12830 kb) [file 259_2020_4758_Fig3_ESM.png]

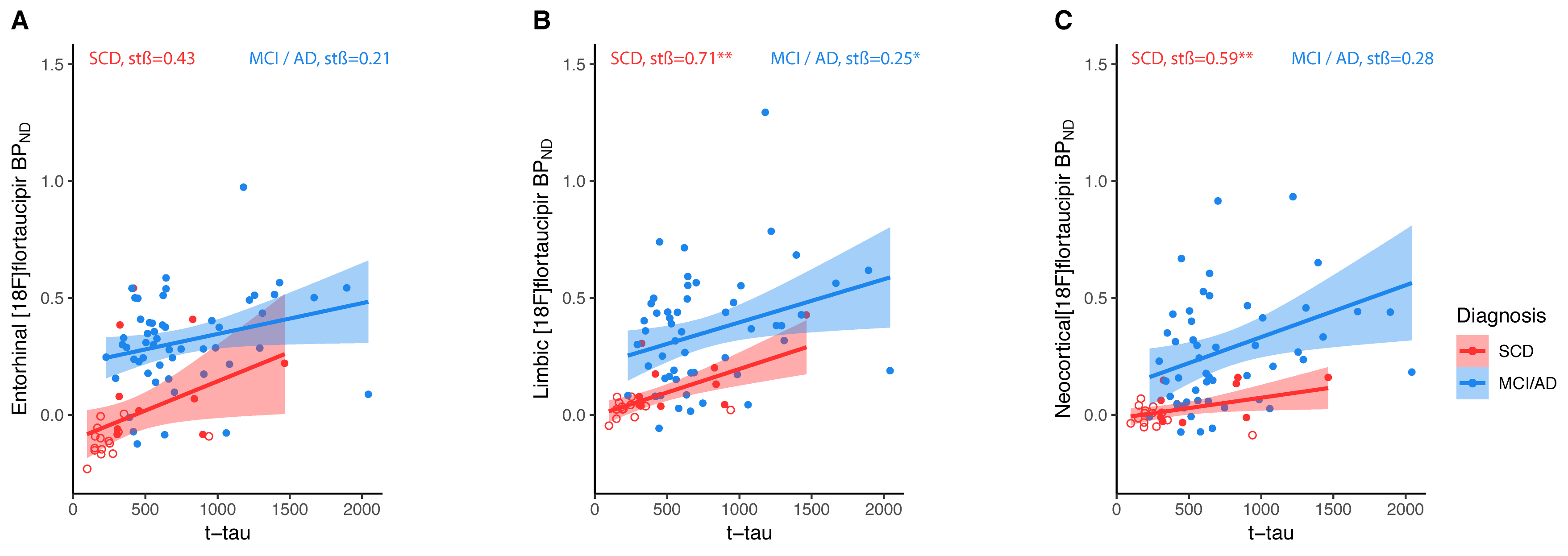

Supplement: Supplementary file 10 — High resolution image (TIF 13503 kb) [file 259_2020_4758_MOESM9_ESM.tif]

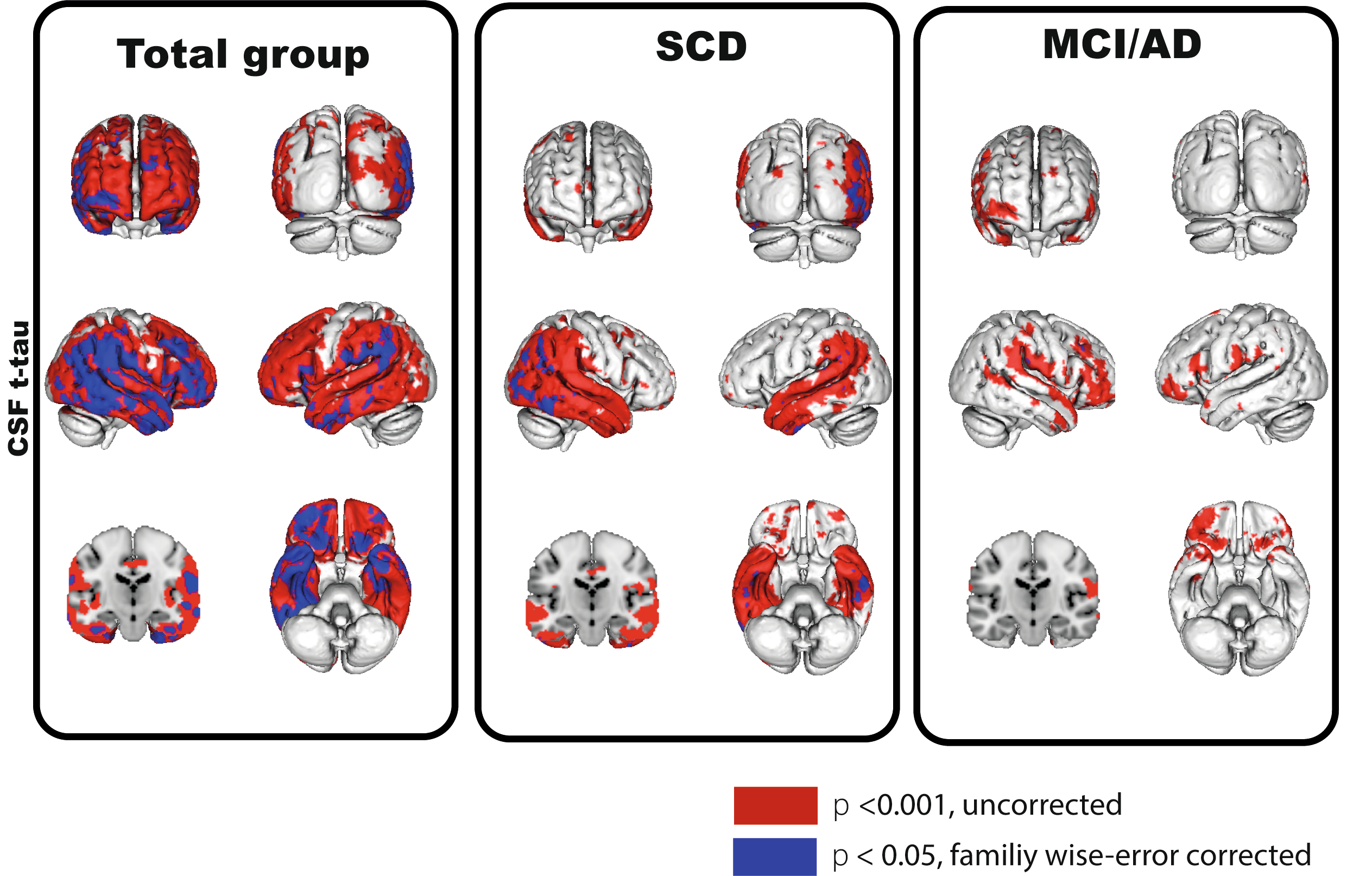

Supplement: Supplementary file 11 — Voxel-wise associations between CSF t-tau and [18F]flortaucipir BPND. Voxel-wise associations are shown using a threshold puncorrected < 0.001(red) and pFWEcorrected < 0.05 (blue) at the voxel level. Contrasts were adjusted for age, sex, and time lag between LP and [18F]flortaucipir PET scan. The associations were assessed in the total sample, within SCD subjects only and within MCI/AD subjects only. (PNG 2198 kb) [file 259_2020_4758_Fig4_ESM.png]

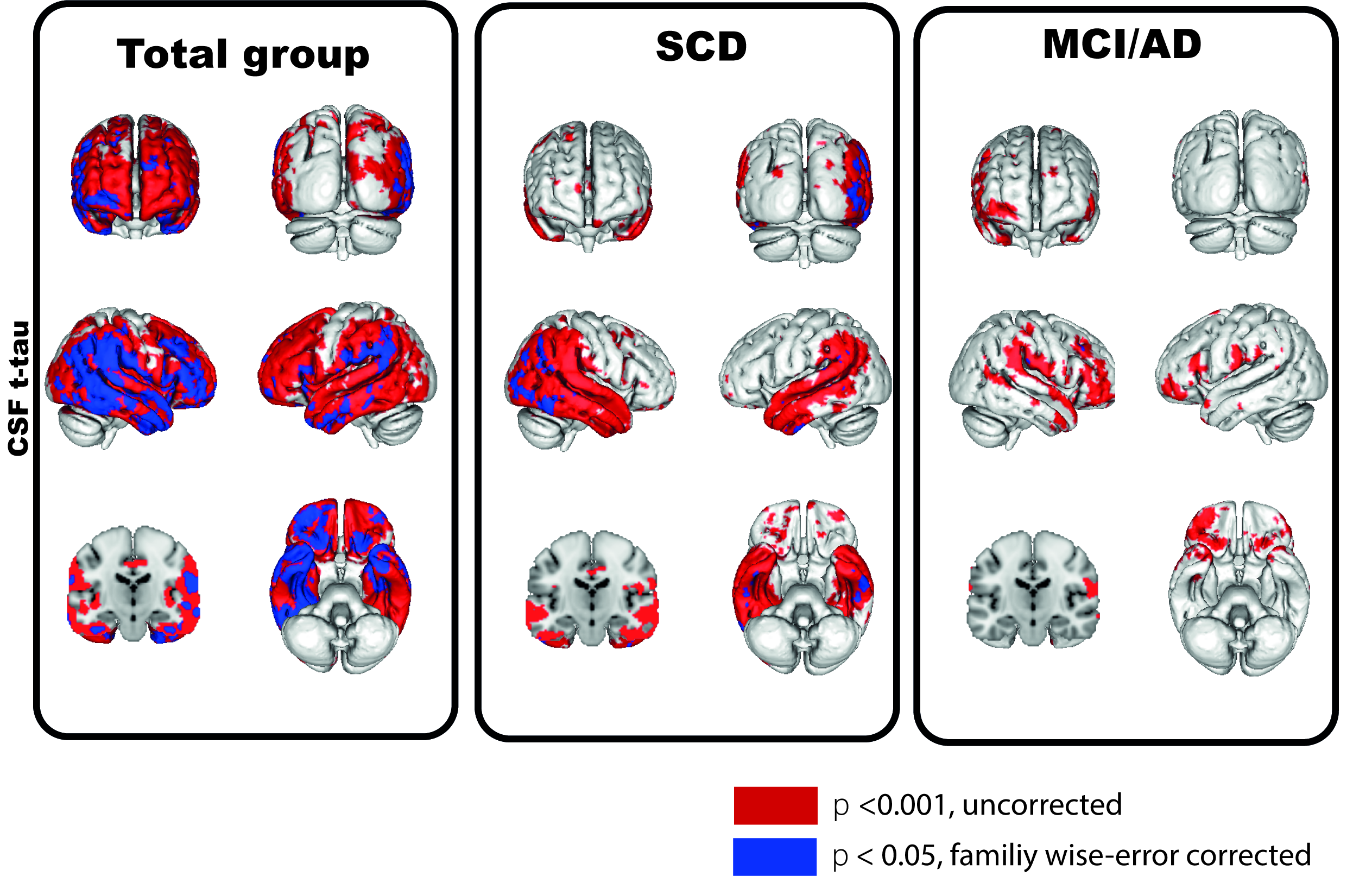

Supplement: Supplementary file 12 — High resolution image (TIF 20022 kb) [file 259_2020_4758_MOESM10_ESM.tif]
